# Conditional Intravesical Recurrence-Free Survival Rate After Radical Nephroureterectomy With Bladder Cuff Excision for Upper Tract Urothelial Carcinoma

**DOI:** 10.3389/fonc.2021.730114

**Published:** 2021-10-07

**Authors:** Jae Hoon Chung, Wan Song, Minyong Kang, Hwang Gyun Jeon, Byong Chang Jeong, Seong IL Seo, Seong Soo Jeon, Hyun Moo Lee, Hyun Hwan Sung

**Affiliations:** Department of Urology, Samsung Medical Center, Sungkyunkwan University School of Medicine, Seoul, South Korea

**Keywords:** urothelium cancer, nephroureterectomy, bladder, recurrence, risk

## Abstract

**Background:**

To evaluate the conditional intravesical recurrence (IVR)–free (IVRF) survival rate in patients with upper tract urothelial carcinoma (UTUC) who had no history of bladder cancer and no concomitant bladder cancer. Hence, we aimed to analyze a relatively large number of patients with UTUC who underwent radical nephroureterectomy with bladder cuff excision (RNUx).

**Methods:**

We retrospectively analyzed the data of 1,095 patients with UTUC who underwent RNUx. Their baseline characteristics, bladder tumor history, and UTUC features were analyzed to evaluate oncological outcomes. To determine the factors affecting IVR, surgical modality, use of preoperative ureteroscopy, TNM stage, and pathological outcomes were evaluated. Multivariable Cox regression analyses were performed to evaluate the factors affecting IVR. Conditional IVRF survival rate was analyzed using Kaplan–Meier curves.

**Results:**

Among the 1,095 patients, 462 patients developed IVR, and the mean time to the development of IVR was 13.08 ± 0.84 months after RNUx. A total of 30.74% of patients with IVR and 15.32% of those without IVR had a history of bladder cancer (p < 0.001). Multivariable analysis showed that a history of bladder cancer, multifocal tumors, use of preoperative ureteroscopy, extravesical bladder cuffing method, lymph node involvement, positive surgical margins, and use of adjuvant chemotherapy were determined to be risk factors for IVR. The conditional IVRF rate was 74.0% at 12 months after RNUx, 87.1% at 24 months after RNUx, 93.6% at 36 months after RNUx, and 97.3% at 60 months after RNUx. The median IVRF survival period was 133.00 months for all patients. In patients with IVRF at 24 months after RNUx, only ureteroscopy was an independent risk factor for IVR [hazard ratio (HR) 1.945, p = 0.040]. In patients with IVRF at ≥36 months, there was no significant factor affecting IVR.

**Conclusions:**

Active IVR assessment is required until 36 months after RNUx. In addition, patient education and regular screening tests, such as urine analysis and cytology, are required for patients with IVRF for ≥36 months.

## Introduction

Upper tract urothelial carcinoma (UTUC) is a urothelial cancer that occurs in the ureter or pelvocaliceal system. It has an incidence of 1–2 cases per 100,000 patients (1, 2). Recently, the incidence of UTUC has been increasing with the development of diagnostic techniques, such as radiological and endoscopic techniques (3). Although the etiology of UTUC remains unclear, cigarette smoking, herbal medicines, chronic infection, and occupational carcinogenesis are known risk factors for UTUC (3). Its gold standard treatment is radical nephroureterectomy with bladder cuff excision (RNUx), which has excellent oncological outcomes (4). However, an important disadvantage of RNUx is intravesical recurrence (IVR), which occurs in 15%–50% of patients after RNUx (5, 6). Diagnosis of IVR after RNUx is important because additional treatments, such as surgery and chemotherapy, due to bladder cancer are required. Moreover, it may affect the prognosis of UTUC (7).

UTUC and bladder cancer have the same histological subtype and urothelial composition. Nevertheless, the two have embryological, epidemiological, and molecular differences (8). Because of these common features, approximately 13% of patients with UTUC have a history of bladder cancer, and approximately 9% of UTUC cases are diagnosed at the same time as bladder cancer (9). Furthermore, there is a high IVR rate after RNUx (10).

Accurate assessment of risk factors for IVR after RNUx is important in reducing unnecessary examinations and treatments for bladder cancer (11). Several previous studies were conducted with the aim of evaluating and preventing IVR, which occurred after RNUx. In these studies, tumor location, cancer stage, grade, and sex were reported as risk factors for IVR after RNUx (7, 12, 13). However, there are limited guidelines on the follow-up protocol for IVR. Despite the limited evidence, IVR occurs most commonly within 12 months after RNUx (14). Moreover, there are insufficient reports on the probability of recurrence of IVR or follow-up when there is no recurrence within 12 months or no IVR for a specific period. In particular, there is no report of IVR-free (IVRF) survival for patients who did not have a history of bladder cancer or concomitant bladder cancer and UTUC.

Therefore, this study aimed to evaluate the conditional IVRF survival rate of patients with UTUC who had no history of bladder cancer and no concomitant bladder cancer and to determine the incidence and risk factors for IVR by analyzing a relatively large number of patients who underwent RNUx for UTUC.

## Patients and Methods

### Patients and Clinicopathological Parameters

We retrospectively analyzed 1,095 patients who underwent RNUx for UTUC at a single medical center from 1994 to 2018. All patients underwent the standard open or minimal invasive surgery, and specimens were collected for further examination. Preoperative ureteroscopy (URS) was not performed routinely. Lymph node (LN) dissection was not routinely performed on all patients. Nevertheless, it was performed only if LN invasion was suspected based on the radiological evaluation. To evaluate the risk factors for IVR, the 1,095 patients were divided into two groups: patients with IVR (n = 462) and those without IVR (n = 633).

Among the 1,095 patients, 856 patients without a history of bladder cancer and without concomitant bladder cancer were assessed for additional analysis for conditional IVRF survival. To evaluate IVRF survival, these 856 patients were divided into two groups: 536 patients with IVR and 320 without IVR.

Baseline characteristics were evaluated, including age, sex, body mass index (BMI), American Society of Anesthesiologists (ASA) score, smoking history, bladder tumor history, and underlying disease including hypertension (HTN) and diabetes mellitus (DM). In addition, the location, laterality, and multifocality of UTUC were analyzed to evaluate oncological outcomes. Surgical modality, approach methods, use of preoperative URS, TNM stage, and pathological outcomes were subsequently evaluated. Tumors were staged according to the 2010 American Joint Committee on Cancer/International Union Against Cancer TNM classification (15). To evaluate conditional IVRF survival, subanalysis was performed in patients with IVRF for 6, 12, 24, 36, 48, and 60 months.

### Statistical Analysis

The groups were compared using the chi-square test for categorical variables and Student’s t-test for continuous variables. Multivariable Cox regression analyses were performed to identify risk factors for IVR, and the IVRF survival rate was analyzed using the Kaplan–Meier curves. Statistical analyses were conducted using the SPSS^®^, version 21.0. For all two-sided tests, p-value <0.05 was considered statistically significant.

### Ethics Statement

The study was performed in agreement with the applicable laws and regulations, good clinical practices, and ethical principles described in the Declaration of Helsinki. The Institutional Review Board of the Samsung Medical Center approved the present study (IRB No. 2019-09-115-002). Requirement for informed consent was waived by the Board.

## Results

Among the 1,095 patients, 462 patients developed IVR. The mean age of patients with and without IVR was 64.88 ± 10.35 and 65.92 ± 11.28 years, respectively (p = 0.120). The prevalence rates of HTN and DM were 48.05% and 22.29%, respectively, in the IVR group and 41.71% and 16.43%, respectively, in the without IVR group (HTN: p = 0.037, DM: p = 0.014). A total of 30.74% patients in the IVR group and 15.32% in the without IVR group had a history of bladder cancer (p < 0.001). Multifocal tumors were observed in 25.54% patients in the IVR group and 22.75% in the without IVR group (p = 0.003).

Cuffing was performed using the intravesical approach in 51.95% patients in the IVR group and 60.19% in the without IVR group (p = 0.007). Preoperative URS was performed in 59.53% patients in the IVR group and 44.39% in the without IVR group (p < 0.001). Adjuvant chemotherapy was performed in 81 patients (17.53%) in the IVR group and 159 patients (25.12%) in the without IVR group (p < 0.001) (**Table 1**).

**Table 1:** Baseline characteristics and operative and pathological outcomes.

	IVR group, *n* = 462	Non-IVR group, *n* = 633	p-value
Age, years	64.88 ± 10.35	65.92 ± 11.28	0.120
Sex, male, n (%)	352 (76.19)	454 (71.72)	0.098^a^
BMI, kg/m^2^	24.40 ± 3.32	24.21 ± 3.00	0.322
ASA score, ≤2, n (%)	415 (89.83)	572 (90.36)	0.116^a^
HTN	222 (48.05)	264 (41.71)	0.037^a^
DM	103 (22.29)	104 (16.43)	0.014^a^
Smoking, never-smoker, n (%)	215 (46.54)	297 (46.92)	0.771^a^
Ex-smoker	134 (29.00)	176 (27.80)	
Current smoker	111 (24.03)	154 (24.33)	
Gross hematuria	369 (79.87)	475 (75.04)	0.060^a^
History of bladder tumor	142 (30.74)	97 (15.32)	<0.001^a^
Laterality			0.159^a^
Left	239 (51.73)	360 (56.87)	
Right	223 (48.27)	272 (42.97)	
Tumor location			0.057^a^
Renal pelvis	188 (40.69)	302 (47.71)	
Ureter	217 (46.97)	269 (42.50)	
Both renal pelvis and ureter	57 (12.34)	62 (9.79)	
Multifocal tumor, n (%)	118 (25.54)	114 (22.75)	0.003^a^
Surgical modality, n (%)			0.395^a^
MIS	242 (52.38)	348 (54.98)	
Open	220 (47.62)	285 (45.02)	
Extravesical bladder cuffing, n (%)	222 (48.05)	252 (39.81)	0.007^a^
Preoperative ureteroscopy, n (%)	275 (59.53)	281(44.39)	<0.001^a^
pT stage, n (%)			<0.001^a^
pTa	62 (13.42)	73 (11.53)	
pT1	147 (31.82)	175 (27.65)	
pT2	94 (20.35)	90 (14.22)	
pT3-pT4	159 (34.42)	295 (46.60)	
Grade, n (%)			0.002^a^
Grade 3	191 (41.34)	315 (49.76)	
Concomitant CIS, n (%)	52 (11.26)	70 (11.06)	0.906^a^
Lymph node, n (%)			<0.001^a^
pN0	97 (21.00)	179 (28.28)	
pN1	26 (5.63)	75 (11.85)	
pNx	339 (73.38)	379 (59.87)	
Tumor size, cm	3.81 ± 3.15	3.91 ± 2.84	0.578
Lymphovascular invasion, n (%)	78 (16.88)	133 (21.01)	0.087^a^
Surgical margin positive, n (%)	26 (5.63)	22 (3.48)	0.086^a^
Adjuvant chemotherapy, n (%)	81 (17.53)	159 (25.12)	0.003^a^
Follow-up, months	60.14 ± 51.68	44.37 ± 45.54	<0.001

IVR, intravesical recurrence; BMI, body mass index; ASA, American Society of Anesthesiologists; HTN, hypertension; DM, diabetes mellitus; MIS, minimal invasive surgery; CIS, carcinoma in situ.

Student’s t-test, ^a^chi-square test.

**Figure 1:**
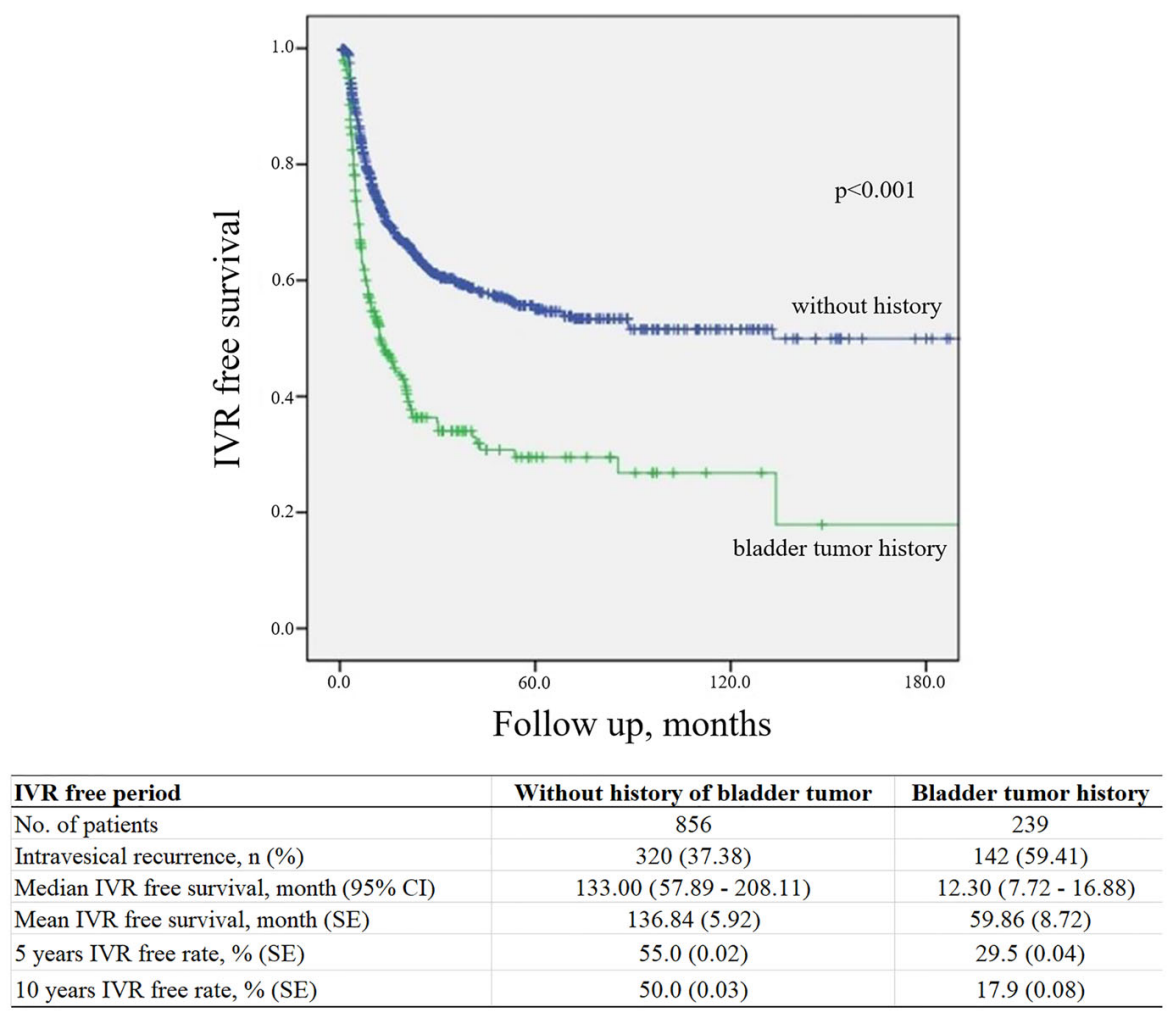
Kaplan–Meier curve for intravesical tumor recurrence according to a history of bladder cancer. IVR, intravesical recurrence; CI, confidence interval; SE, standard error.

IVR occurred at a mean period of 13.08 (interquartile range, 3.97–14.05) months after RNUx. The median IVRF survival period was 12.30 (95% CI, 7.72–16.88) months in patients with a history of bladder cancer or concomitant bladder cancer and 133.00 (95% CI, 57.89–208.11) months in patients without such a history (p < 0.001) (**Figure 1**). Multivariable analysis revealed a history of bladder cancer [hazard ratio (HR) 2.409, p < 0.001], a multifocal tumor (HR 1.348, p = 0.008), use of preoperative URS (HR 1.733, p < 0.001), extravesical bladder cuffing (HR 1.408, p = 0.009), LN involvement (HR 2.121, p = 0.004), positive surgical margins (HR 1.553, p = 0.026), and adjuvant chemotherapy (HR 0.759, p = 0.033) to be risk factors for IVR (**Table 2**).

**Table 2:** Results of the univariable and multivariable Cox regression analyses.

	Univariable			Multivariable		
	HR	95% CI	p-value	HR	95% CI	p-value
Age (continuous)	1.016	1.007–1.025	0.001	1.000	0.988–1.013	0.959
Sex, male	1.108	0.894–1.373	0.348			
BMI	0.985	0.957–1.014	0.302			
DM	1.424	1.143–1.773	0.002	1.279	0.912–1.794	0.154
HTN	1.248	1.040–1.498	0.017	1.294	0.975–1.717	0.074
Gross hematuria	1.148	0.914–1.441	0.235			
Smoking	0.949	0.851–1.057	0.342			
Bladder cancer history	2.184	1.791–2.665	<0.001	2.409	1.761–3.297	<0.001
ASA score	1.229	1.050–1.438	0.010	1.112	0.861–1.436	0.417
Laterality	0.868	0.723–1.041	0.127			
Location						
Renal pelvis	1	reference	–			
Ureter	1.211	0.996–1.473	0.055			
Both	1.487	1.108–1.997	0.008	1.055	0.610–1.824	0.849
Multifocality	1.503	1.219–1.853	<0.001	1.348	1.082–1.678	0.008
Preoperative URS	1.597	1.325–1.925	<0.001	1.733	1.338–2.244	<0.001
Operation modality						
MIS	0.859	0.454–1.624	0.640			
Bladder cuffing, extravesical	1.320	1.099–1.585	0.003	1.408	1.090–1.818	0.009
pT stage						
pTa	1	reference	–			
pT1	1.164	0.867–1.561	0.312			
pT2	1.311	1.047–1.641	0.018			
pT3+4	1.492	1.156–1.926	0.002	1.245	0.958–1.619	0.102
Grade, low grade	0.902	0.781–1.041	0.158			
Concomitant CIS	0.977	0.743–1.285	0.870			
Lymph node positive						
pN0	1	reference	–			
pN1	1.651	1.238–2.200	0.001	2.121	1.274–3.532	0.004
pNx	0.640	0.384–1.065	0.086			
Tumor size	1.009	0.975–1.044	0.602			
Margin positive	2.009	1.350–2.988	<0.001	1.553	0.819–2.947	0.026
Lymphovascular invasion	0.927	0.727–1.183	0.545			
Adjuvant chemotherapy	0.697	0.548–0.886	0.003	0.759	0.589–0.979	0.033

HR, hazard ratio; CI, confidence interval; BMI, body mass index; DM, diabetes mellitus; HTN, hypertension; CVA, cerebrovascular accident; ASA, American Society of Anesthesiologists; URS, ureteroscopy; CIS, carcinoma in situ.

### Conditional IVRF Survival and Risk Factors for IVR in UTUC Patients Without a History of Bladder Cancer or Concomitant Bladder Cancer

**Figure 2:**
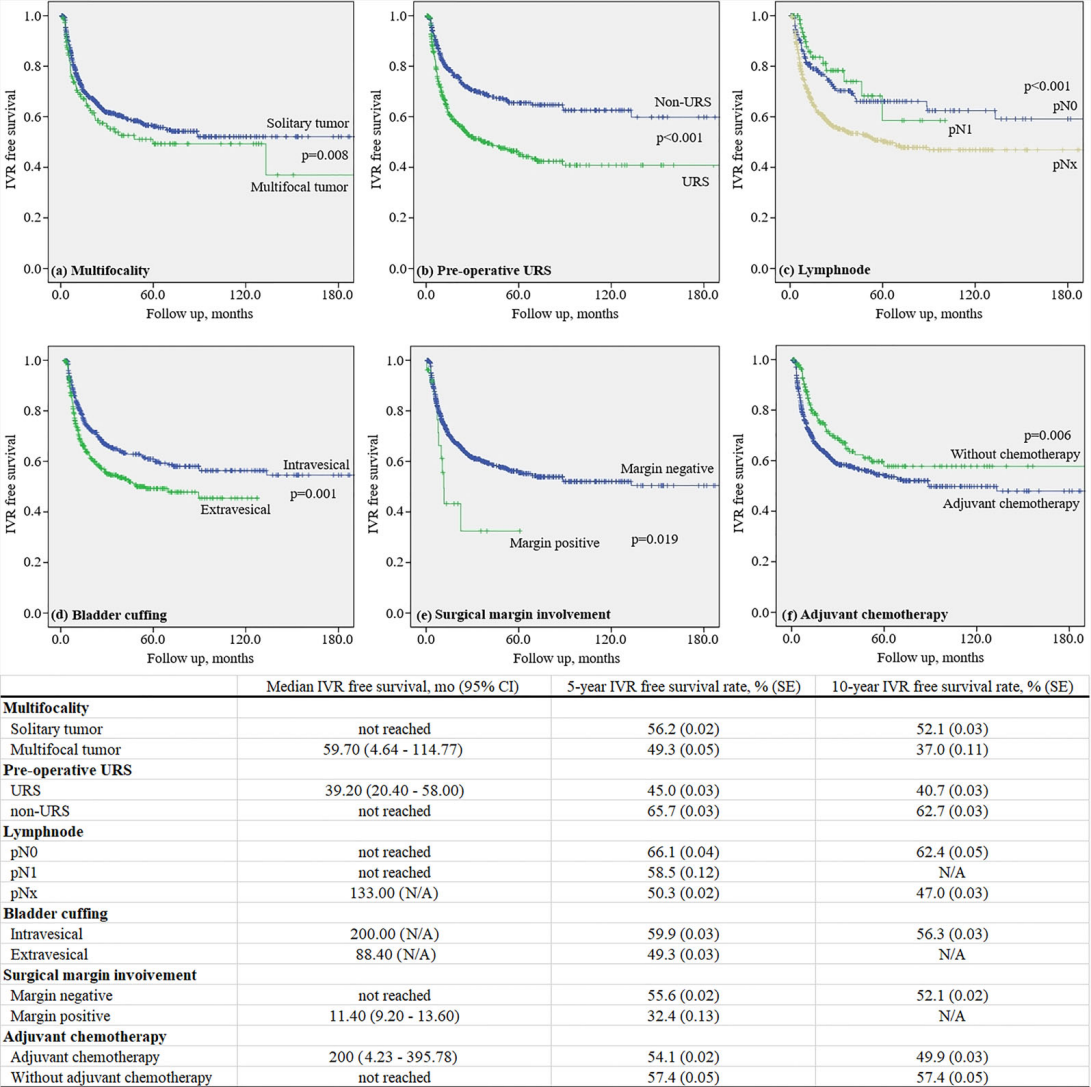
Kaplan–Meier curve for intravesical recurrence in patients without a history of bladder tumor.

The mean 5-year IVRF survival rate after RNUx was 49.3% ± 0.05% for multifocal tumors and 56.2% ± 0.02% for solitary tumors (p = 0.008). The mean 5-year IVRF survival rate after RNUx was 45.0% ± 0.03% for those who underwent preoperative URS and 65.7% ± 0.03% for those who did not (p < 0.001). In the case of LN involvement, the mean 5-year IVRF survival rate after RNU/Bladder cuff excision (BCE) was 58.5% ± 0.12% and 66.1% ± 0.04% for patients with and without involvement, respectively (p < 0.001). At 5 years after RNUx, the mean IVRF survival rate of the intravesical bladder cuffing group was 59.9% ± 0.03% and the extravesical bladder cuffing group was 49.3% ± 0.03% (p = 0.001) (**Figure 2**).

**Figure 3:**
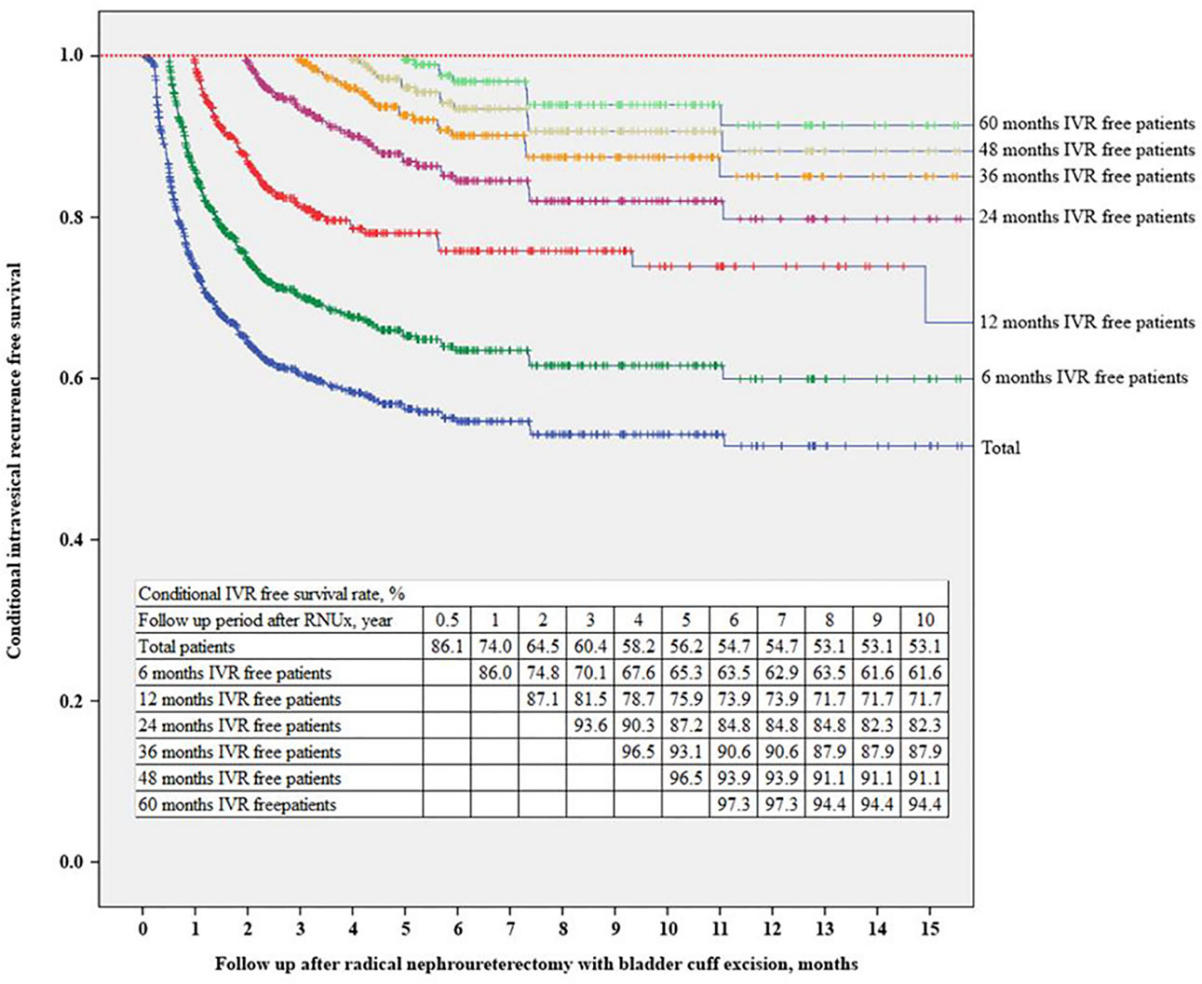
Conditional intravesical recurrence-free survival. IVR, intravesical recurrence; RNUx, radical nephroureterectomy with bladder cuff excision.

The IVRF rate was 74.0% at 12 months after RNUx, 87.1% at 24 months after RNUx, 93.6% at 36 months after RNUx, and 97.3% at 60 months after RNUx (**Figure 3**). The mean IVRF survival period was 136.84 months for all patients, 156.24 months for those with 6-month IVRF, 175.38 months for those with 12-month IVRF, 189.14 months for those with 36-month IVRF, and 178.21 months for those with 60-month IVRF (**Table 3**).

**Table 3:** Conditional intravesical recurrence-free survival after radical nephroureterectomy with bladder cuff excision.

Conditional IVR-free period	Total	0.5 year	1 year	2 years	3 years	4 years	5 years
No. of patients	856	696	544	381	285	228	174
Intravesical recurrence, n (%)	320 (37.38)	205 (29.45)	111 (20.40)	47 (12.34)	25 (8.77)	16 (7.02)	9 (5.17)
Median IVR-free survival, months (95% CI)	133.00 (57.89–208.11)	not reached	not reached	not reached	not reached	not reached	not reached
Mean IVR-free survival, months (SE)	136.84 (5.92)	156.24 (6.34)	175.38 (6.83)	188.67 (7.07)	189.14 (7.03)	183.84 (6.92)	178.21 (6.73)
5 years IVR-free survival rate, % (SE)^*^	56.2 (0.02)	63.5 (0.02)	73.9 (0.02)	84.8 (0.02)	87.9 (0.03)	91.1 (0.02)	94.4 (0.02)
10 years IVR-free survival rate, % (SE)^*^	53.1 (0.02)	61.6 (0.02)	71.7 (0.03)	80.1 (0.03)	85.5 (0.03)	88.7 (0.03)	91.9 (0.03)

CI, confidence interval; SE, standard error.

^*^After conditional IVR-free period.

Multivariable Cox regression analysis showed that DM (HR 1.589, p = 0.017), gross hematuria (HR 1.744, p = 0.003), URS (HR 2.207, p < 0.001), and extravesical bladder cuff excision (HR 1.614, p = 0.002) were found to be risk factors for IVR. In patients with 24-month IVRF survival rate after RNUx, only URS was found to be an independent risk factor for IVR (HR 1.945, p = 0.040). In patients with ≥36-month IVRF survival rate, there was no significant risk factor for IVR (**Table 4**).

**Table 4:** Risk factors for IVR in patients with UTUC who had no history of bladder cancer.

	Total			0.5 year IVRF			1 year IVRF			2 years IVRF		
	HR	95% CI	p-value	HR	95% CI	p-value	HR	95% CI	p-value	HR	95% CI	p-value
Diabetes mellitus	1.589	1.088–2.323	0.017	1.281	0.814–2.017	0.284	1.105	0.609–2.002	0.743	1.173	0.484–2.841	0.724
Gross hematuria	1.744	1.209–2.515	0.003	2.258	1.425–3.580	0.001	2.299	1.252–4.219	0.007	2.083	0.881–4.926	0.095
Ureteroscopy	2.207	1.633–2.985	<0.001	2.14	1.502–3.049	<0.001	2.082	1.324–3.274	0.001	1.945	1.030–3.672	0.040
Bladder cuffing, extravesical	1.614	1.193–2.183	0.002	1.557	1.091–2.220	0.015	1.336	0.847–2.105	0.213	1.208	0.624–2.341	0.575
Grade	0.77	0.589–1.007	0.057	0.643	0.466–0.887	0.007	0.595	0.394–0.900	0.014	0.683	0.384–1.215	0.194
Adjuvant chemotherapy	0.746	0.510–1.092	0.131	1.253	0.819–1.917	0.298	1.407	0.808–2.448	0.227	1.671	0.773–3.612	0.192
	**3 years IVRF**			**4 years IVRF**			**5 years IVRF**					
	**HR**	**95% CI**	**p-value**	**HR**	**95% CI**	**p-value**	**HR**	**95% CI**	**p-value**			
Diabetes mellitus	1.237	0.337–4.535	0.749	2.213	0.563–8.704	0.255	1.137	0.126–10.294	0.909			
Gross hematuria	1.088	0.408–2.903	0.866	0.638	0.201–2.018	0.444	0.945	0.168–5.309	0.948			
Ureteroscopy	1.688	0.724–3.935	0.225	1.548	0.532–4.505	0.422	1.632	0.388–6.861	0.504			
Bladder cuffing, extravesical	0.858	0.349–2.109	0.738	0.427	0.112–1.634	0.214	0.79	0.139–4.509	0.791			
Grade	0.717	0.332–1.549	0.398	0.816	0.311–2.144	0.680	0.848	0.228–3.160	0.806			
Adjuvant chemotherapy	1.493	0.526–4.242	0.452	0.991	0.248–3.967	0.991	0.618	0.067–5.708	0.671			

Multivariable Cox regression analysis.

IVRF, intravesical recurrence-free; HR, hazard ratio; CI, confidence interval.

## Discussion

In this study, the risk factors for IVR after RNUx in patients with UTUC are discussed. Moreover, it was confirmed that there were no significant UTUC-related risk factors for IVR among patients who had ≥36-month IVRF survival rate. However, it was confirmed that approximately 5% of patients with an IVRF period of ≥36 months developed IVR.

After RNUx for UTUC, the cause of IVR is not clear yet. However, the possible mechanisms of IVR include the field theory that UTUC is exposed to the urothelium to generate IVR (16) and the intraluminal tumor seeding theory that the cancer cells in the upper tract reach the bladder *via* the urinary stream (17). If URS is performed before RNUx, the intraluminal pressure of the renal pelvis increases and tumor manipulation occurs, resulting in increased intraluminal tumor seeding and eventually increased IVR (18, 19). In the present study, it was found that URS before RNUx was a risk factor for IVR in patients with 0–24-month IVRF survival rate. The efficacy of URS before surgery remains controversial (20, 21). However, reducing the use of preoperative URS based on radiological imaging and laboratory examinations, such as urine analysis and cytology, could improve the IVR rate.

In a recent meta-analysis, the predictors of IVR were classified into three categories: patient-specific factors, tumor-specific factors, and treatment-specific factors (22). In this study, patient-specific factors included male sex, bladder cancer history, and chronic kidney disease; tumor-specific factors included positive urinary cytology, ureteral location, multifocality, invasive pT stage, and necrosis. Furthermore, treatment-specific factors included laparoscopic surgery, extravesical bladder cuff excision, and positive surgical margins. In the present study, bladder cancer history, preoperative URS, multifocality, LN invasion, bladder cuffing, and surgical margin involvement were identified as risk factors for IVR after RNUx. The present study was also conducted in patients with no history of bladder cancer. In these patients, DM, gross hematuria, URS, and extravesical bladder cuff excision were evaluated as predictive factors of IVR after RNUx possibly owing to the difference between the groups. However, it is clear that avoiding preoperative URS and extravesical bladder cuff excision can reduce IVR.

Previous studies reported that DM is an independent risk factor for IVR (23, 24). Moreover, a previous study reported that DM corresponded to a poor prognosis in patients with UTUC. Chronic exposure to hyperinsulinemia or hyperglycemia is a possible factor that induced tumor cell proliferation (25, 26). Hashimoto et al. (27) reported that gross hematuria was a significant risk factor for IVR. They suggested that hemorrhagic tumor cells could be seeded easily in the mucosal epithelium. Nevertheless, the methods of management for bladder cuff remain controversial. Some studies reported that the method of bladder cuff excision was not associated with the IVR rate (28, 29). However, when intramural ureter was not appropriately resected, the reported recurrence rate was 33%–75% (30).

Recently, Katims et al. (31) reported the risk factors for IVR after minimally invasive RNUx. They reported that IVR occurred in 22.7% of patients who underwent RNUx. In addition, URS, transurethral resection of the bladder cuff and positive surgical margin were suggested to be risk factors for IVR. In the present study, 37.38% of patients without prior or concurrent bladder cancer developed IVR. The different cancer stage of the enrolled patients might have affected higher IVR rate.

Due to concerns about IVR, bladder examination, including cystoscope and urinary cytology, is recommended for 5 years after RNUx for UTUC (4). However, due to the lack of studies, optimal follow-up strategies for IVR after RNUx cannot be concluded (32, 33). Recently, Shigeta et al. (34) reported on conditional IVRF survival after RNUx of the 364 patients with Ta-T3 UTUC. According to this study, IVR was identified in 48.4% patients; the 5-year conditional IVRF survival rate increased from 41.5% to 60.5%, 73.4%, 79.5%, and 96.7% in patients with 1-, 2-, 3-, and 4-year IVRF survival rate, respectively. In the present study, each conditional IVRF survival was evaluated for patients with 0.5-, 1-, 2-, 3-, 4-, and 5-year IVRF survival rate. Our results are consistent with those of a previous study. Moreover, gross hematuria, URS, retroperitoneal approach, and a low grade were identified as risk factors for IVR in patients with 1-year IVRF survival rate, and only URS was evaluated as a risk factor for IVR in patients with 2-year IVRF survival rate. In addition, for patients with >3-year IVRF survival rate, these risk factors were deemed to have no significant effect on IVR. However, IVR occurred in 13.1% of patients with 3-year IVRF survival rate, and only 5.6% of patients with 5-year IVRF survival rate were diagnosed with IVR. Although these could not be clearly identified as IVR associated with UTUC, it was found to be a higher incidence than that in the general population. Therefore, patients with UTUC require IVR follow-up even following 5 years after RNUx.

Conditional survival analysis is a method of assessing additional survival at a specific time point after initial diagnosis or treatment (35). It is widely performed in cancer research because it can transmit additional important and diverse information during follow-up (36, 37). In this study, conditional IVRF survival according to the IVRF period was found in a large number of UTUC patients without a history of bladder cancer. According to the conditional IVRF survival rate in the present study, in the case of patients whose IVRF period is <36 months, the IVR rate after the IVRF period is >10%; hence, active assessment is required until 3 years after RNUx. In addition, since IVR occurs in 3%–5% of patients with an IVRF period of ≥36 months, patient education and screening tests, such as regular urine analysis and cytology, are required.

This study has several limitations. First, it was a retrospective study; thus, fixed criteria for diagnosis, treatment, and patient follow-up could not be used. Second, the indications for the use of URS, lymphadenectomy, and adjuvant chemotherapy were not clear. Although this study could not suggest an optimal follow-up strategy for IVR after RNUx for UTUC, it was considered that follow-up for IVR could be optimized through systematic analysis if additional data would be collected in the future. Moreover, notably, to the best of our knowledge, it is the first large-scale study on the conditional survival of patients with UTUC who had no history of bladder cancer.

## Conclusion

In this study, a history of bladder cancer, multifocal tumors, preoperative URS, LN invasion, extravesical bladder cuffing, and surgical margin involvement were identified as risk factors for IVR. However, in patients with no history of bladder cancer, DM, gross hematuria, preoperative URS, and extravesical bladder cuffing were risk factors. In patients whose IVRF period is <3 years, the IVR rate is ≥10%; hence, active IVR assessment is required until 3 years after RNUx. In addition, since 3%–5% of patients with an IVRF survival period of ≥3 years develop IVR, patient education and regular screening tests, such as urine analysis and cytology, are required for patients with ≥3-year IVRF survival rate.

## Data Availability Statement

The raw data supporting the conclusions of this article will be made available by the authors without undue reservation.

## Ethics Statement

The studies involving human participants were reviewed and approved by Samsung medical center. Written informed consent for participation was not required for this study in accordance with the national legislation and the institutional requirements.

## Author Contributions

HS contributed to the conceptualization. WS, MK, HJ, BJ, SS, SJ, and HL contributed to the methodology. JC contributed to the formal analysis. JC and HS contributed to the data curation. JC contributed to writing–original draft preparation. HS contributed to writing–review and editing and supervision. All authors contributed to the article and approved the submitted version.

## Conflict of Interest

The authors declare that the research was conducted in the absence of any commercial or financial relationships that could be construed as a potential conflict of interest.

## Publisher’s Note

All claims expressed in this article are solely those of the authors and do not necessarily represent those of their affiliated organizations, or those of the publisher, the editors and the reviewers. Any product that may be evaluated in this article, or claim that may be made by its manufacturer, is not guaranteed or endorsed by the publisher.
